# The Application of a System of Eye Tracking in Laparoscopic Surgery: A New Didactic Tool to Visual Instructions

**DOI:** 10.3389/fsurg.2021.643611

**Published:** 2021-06-09

**Authors:** Ester Marín-Conesa, Francisco Sánchez-Ferrer, María Dolores Grima-Murcia, María Luisa Sánchez-Ferrer

**Affiliations:** ^1^Department of Neurology, ‘Reina Sofía’ University Hospital of Murcia, Murcia, Spain; ^2^Department of Pediatrics, “San Juan” University Hospital, Miguel Hernandez University, Alicante, Spain; ^3^Anatomical Innovation Service, Bioingineering Institute, Miguel Hernandez University, Alicante, Spain; ^4^Department of Obstetrics and Gynecology of the University Hospital “Virgen de la Arrixaca”, Institute for Biomedical Research of Murcia, IMIB-Arrixaca, University of Murcia, Murcia, Spain

**Keywords:** eye tracking systems, laparoscopic surgery, learning tool, real surgery room, Tobii glasses 2, evaluation tool

## Abstract

**Introduction:** Laparoscopic surgery is an increasingly used technique, but it requires a high degree of learning, and communication between the operating room crew is considerably difficult. The use of eye tracking has been proposed as a didactic and evaluation tool in several settings, including in laparoscopy in simulators.

**Objectives:** This study aimed to evaluate the usefulness of the use of eye tracking systems (Tobii glasses 2) in laparoscopic surgery as a didactic and assessment tool to improve communication in the operating room and improve patients' security.

**Methodology:** An anonymous survey was sent to the students and medical teachers of a faculty of medicine and practicing doctors and residents. The message contained an explanation about the use of the Tobii glasses, a link to watch the video showing its use in a laparoscopic surgery, and the survey to complete after watching the video.

**Results:** The survey was answered by 113 participants (51.3% medical students, 27.4% medical teachers, 18.6% practicing doctors, and 2.7% medicine residents). Eighty-three percent agreed with the usefulness of the “Tobii glasses” in the operating room for improving communication between the main surgeon and the assistant, for learning complex surgery techniques, for obtaining didactic videos, and for indicating anatomical structures. The item scored worst was the price of the glasses.

**Conclusions:** It is possible to record and project expert gaze patterns in the operating room in real time using the Tobii glasses. This device allows improving communication among the surgical crew and the learning of residents and also improving the security of surgical patients.

## Introduction

Laparoscopy is a surgical technique increasingly used due to the advantages it offers, such as the fast recovery of patients. However, it presents as disadvantages the requirement of great technical learning and the acquisition of psychomotor skills that are different from those used in open surgery (1). Besides, during the performance of laparoscopic surgery, as the surgeon cannot point to the monitor due to wearing a sterile suit and gloves and having to handle the laparoscopic instruments, when he gives verbal instructions, uncertainty may arise about the exact point to which he is referring, which hinders learning and may also be difficult for communication among the various members of the surgical team (2). Likewise, in laparoscopic surgery, the main surgeon often operates the surgical instruments while the assistant controls the laparoscopic camera and other secondary instruments. As a result of this, it is important for the surgical team to focus on the same target in order to proceed efficiently (3). As a consequence of this need for fast and quality learning, new training techniques have been proposed based on gaze control (through the use of eye tracking in real time) for learning in laparoscopic surgery.

Eye tracking (eye movements) has been recorded using stationary cameras or cameras fitted to normal glasses. These glasses can record corneal reflection to track the pupils' position in order to map gaze focusing in the video recording in real time. Eye tracking is been used in a wide range of areas, such as market research and advertising testing, the design of web pages, eye control for accessibility (very useful for quadriplegics, for example), psychology and vision research, gaze interaction and car assistant systems and medical research, and in diagnostics and rehabilitation (4). On the other hand, the importance of the strategic behavior of the gaze in the optimization of motor control has been shown (5). Improved learning has been demonstrated when students are shown the exact points on which an expert stops his gaze, which means to follow their gaze pattern, which has been achieved through the use of eye-tracking technology (6, 7). Regarding the health field, we can find its application in many fields, such as in radiology. Dempere-Marco et al. (8) trained novices by showing them the visual search pattern that expert radiologists had carried out during the evaluation of several CTs, improving their accuracy.

O'Meara et al. (9) used eye tracking to train nursing and paramedicine students while facing emergency simulations, obtaining an improvement in student learning.

Many studies (10–13) have analyzed the gaze patterns of experts and novices and showed that there are differences between both gaze patterns and pupillometry data. Studies (11, 13) that use these differences between beginners and experts as a didactic tool to improve the learning of novices and as a method to evaluate them have been carried out. In surgery, Di Stasi et al. (14) concluded that the metrics of the gaze (entropy of the gaze and speed) were a valid and reliable index for evaluating a workload.

In laparoscopy, eye tracking has been proposed as a didactic and evaluation tool (15). Most of these studies were performed in simulated laparoscopic surgery (Supplementary Table 1). Chetwood et al. (2) conducted an interesting research on laparoscopic simulators using the Tobii 1750, 17-in. computer monitor device (Tobii Technology AB, Danderyd, Sweden). The subjects performed the laparoscopic task, receiving instructions from an expert at the same time, which were different according to the group. Three groups were formed with different instructions: (1) verbal queues, (2) a cursor reflecting the supervisor's eye gaze, and (3) both verbal cue and eye gaze. Completion times and the number of errors were significantly reduced when the eye gaze instruction was employed. In addition, the time taken for the subject to correctly focus on the target (latency) was significantly reduced.

We found three studies conducted during real laparoscopy, but the analysis of eye tracking was done after the surgical procedure (16–18). We only found one study during real laparoscopy (12) where the authors published their own experiences with performing eye tracking to improve surgery training. Our objectives were to record a video in a real laparoscopy using Tobii glasses and to evaluate with a Likert-type scale whether this didactic tool could improve the learning of students, residents, and practicing doctors through a survey.

## Materials and Methods

We conducted an observational prospective study in April 2017. This study was approved by the Ethics Research Committee of our university and our clinical university hospital (No. 2015-10-5-HCUVA, approved October 26, 2015). Written informed consent was obtained from all participants. All authors have reviewed this manuscript and approved its submission.

### Laparoscopic Procedure Video

Tobii glasses 2 were tested in a real laparoscopic procedure at a university clinical hospital. The operation consisted of right adnexectomy by laparoscopy for ovarian cyst in a postmenopausal patient. A video of this procedure was recorded (see Supplementary Video 1).

### Eye-Tracking System

**Figure 1:**
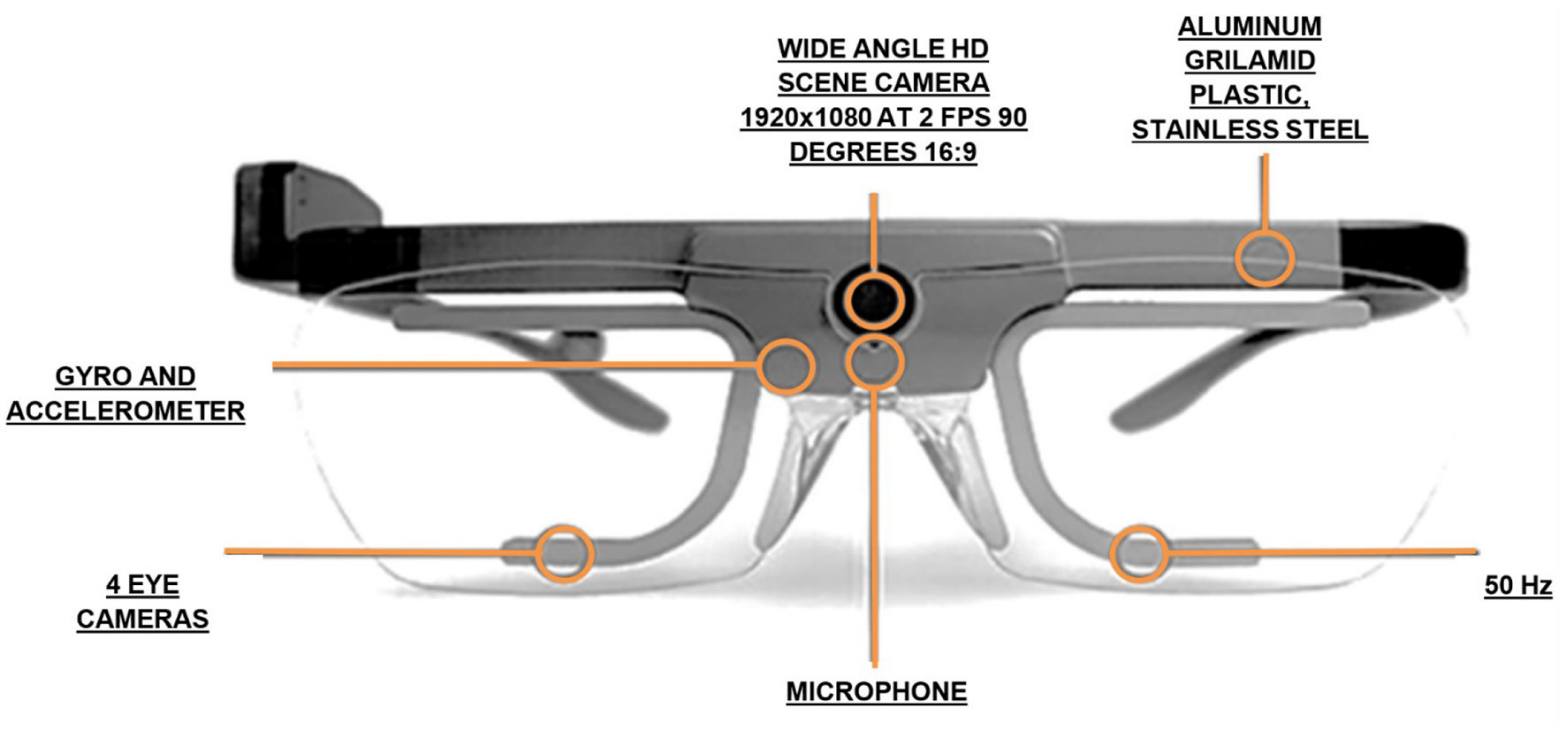
Tobii glasses 2 (technical details).

The device used in the video was Tobii glasses 2 (Figure 1). It is a video-based eye-tracking system that uses infrared light. It is integrated in normal glasses (weight, 45 g), with a horizontal visual field of 160 degrees. The total battery time is 120 min, and the software runs on a Windows 8/8.1 or Windows 7 or 8/8.1 tablet. The connection of the glasses with the tablet is through Wi-Fi. It has a data transfer speed of 50 Hz. Through this connection, images and sounds are transmitted to any computer (with the indicated software) with a delay of <1 s. In addition, it allows free movements of the subject that carries it. The system must be calibrated separately for each participant, with the participants having to look at a calibration card for several seconds. After this, it is possible to start recording the subject's vision through the Tobii glasses. The visual field of the subject is recorded by an HD camera located at the front of the Tobii glasses; at the same time, the areas on which the subject fixes his/her gaze are detected (foveal vision is tracked). During the real-time visualization, a red circle is observed, which represents the foveal vision of the subject, on the recording of the visual field. The ocular tracking performed can be seen both in real time and later (as it is recorded).

### Anonymous Survey

The participants were students and medical teachers of a faculty of medicine and practicing doctors and residents of a university clinical hospital. All were invited through their university e-mails to complete an anonymous online survey about a laparoscopic procedure video showing the use of eye-tracking technology to objectively measure the usefulness of this instrument.

The survey had two parts. The first one consisted of five general items to collect epidemiological data. The second part contained nine items to value different aspects of the video using the Tobii glasses. In this part, a Likert-type scale was used with five possible answers: “Completely agree,” “Agree,” “Undecided,” “Disagree,” and “Completely disagree” (Table 1).

**Table 1:** Survey on laparoscopic applications of Tobii glasses.

*Complete:*
✓ Age: ___ ✓ Gender: ___ (1 = male, 2 = female) ✓ Current status: ___ (1 = medical student, 2 = medical teacher, 3 = practicing doctor, 4 = resident of medicine) ✓ If you are a medical student, indicate your academic year: ___ (1 = 1st year, 2 = 2nd year, 3 = 3rd year, 4 = 4th year, 5 = 5th year, 6 = 6th year) ✓ If you are a doctor, indicate your specialty: ___ (1 = medical specialty, 2 = surgical specialty, 3 = medical–surgical specialty)
*After watching the video about the applications in the operating room of the “Tobii glasses,” you think:* *(1 = Completely agree, 2 = Agree, 3 = Undecided, 4 = Disagree, 5 = Completely disagree)*
1. That it could be a good teaching tool during the performance of laparoscopic surgery by allowing the surgeon who takes them to point out concrete structures in real time on the monitor, in which the image of the laparoscopy is shown. 2. It is a great help for the assistant to let him know exactly where the surgeon is looking. 3. That it seems a good tool to improve communication between the main surgeon and the assistant during laparoscopic surgery by allowing to point out concrete structures on the monitor, providing a “third hand.” 4. That it seems to be a good tool to use in operating rooms during surgical operations, like sometimes, when pointing structures with a clamp, it is not always visible from all angles because necessarily the surgeon himself covers the structures from the assistant or the student. 5. That it could be a good tool for specialization in more complex surgical techniques by allowing the most expert surgeon to signal to the novice surgeon the surgical gestures to be performed during the most complex laparoscopies. 6. That it seems to be a good tool to obtain didactic videos that register the surgeon's visual field and his surgical strategy (the places where he has fixed his gaze and that have allowed him to plan the surgical gestures that he has subsequently executed). He also records the execution of the surgery, noting the structures he is working on. 7. That it seems to be a very useful tool to point out anatomical structures during the teaching of the anatomy applied to the clinic in postgraduate courses, using cadaveric models fixed in Thiel that allows the realization of laparoscopic surgical techniques. 8. That it could be a good tool to evaluate the learning of the residents because it could register the pattern of their gaze and compare it with that of an expert surgeon (the more similar they are, the more surgical skill the resident has). It allows to monitor curves of learning objectively. 9. The current cost of the glasses is approximately 14,000 Euros. Do you think that it is an efficient product for acquisition in the Faculties of Public Medicine and/or university hospitals linked to the effort in the incorporation of educational innovation strategies?

### Procedure and Data Analyses

The survey was sent to the participants through the computer application of our university. The survey message contained an explanation about the use of the Tobii glasses, a link to watch the video showing its use in laparoscopic surgery, and the survey to complete after watching the video. This same application was used to analyze the data. Non-parametric range, median, or range-based procedures were used for analyzing these data as distribution-free methods such as tabulations, frequencies, contingency tables, and chi-square statistics. The results were presented as bar charts.

## Results

**Figure 2:**
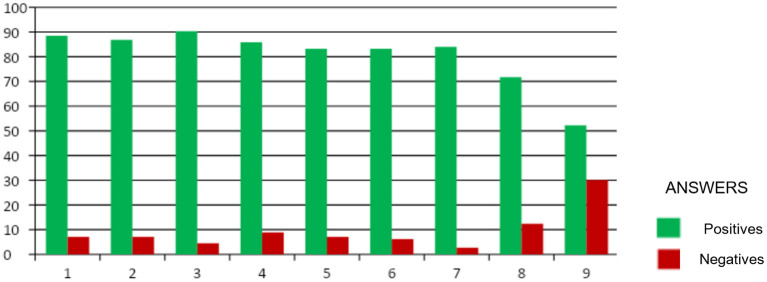
Overall response to the different items of the survey (positive: “Completely agree” and “Agree”; negative: “Disagree” and “Completely disagree”).

A total of 113 surveys were returned. The participants were 51.3% medical students, 27.4% medical teachers, 18.6% practicing doctors, and 2.7% medical residents. Figure 2 shows a graph in which the responses “Completely agree” and “Agree” have been grouped as positive responses and “Disagree” and “Strongly disagree” as negative responses. The results have been described independently of the status (medical student, teacher, practicing physician, or residents). The positive responses grouped into the different items were 88.5% for item 1, 86.8% for item 2, 90.3% for item 3, 85.8% for item 4, 83.2% for item 5, 83.2% for item 6, 84% for item 7, 71.7% for item 8, and 52.2% for item 9. We can see that the overall results of the survey are very satisfactory.

The items for questions 1–7 have received satisfactory answers above 83%, being the best rated. It deals with questions regarding the usefulness of the Tobii glasses in the operating room in the teaching of residents, in the improvement of communication between the main surgeon and the assistant, providing a “third hand,” in the learning of techniques for complex surgeries, and in obtaining didactic videos and indicating anatomical structures in real laparoscopy. On the other hand, the item with the worst score was number 9, with 52.2% positive responses, which was in relation to the price of these glasses.

## Discussion

Our group is a pioneer in the use of eye tracking in real time during a real laparoscopy, which allowed the surgeon to use his/her gaze as a pointer, thanks to the option of projecting the foveal fixation of the expert surgeon in real time, thus being able to indicate the exact point to which he/she is referring without endangering the sterility of the surgical field. This sets the possibility of learning based on gaze control in the surgery room at the same time that the surgeon operates, improving the communication among the different members of the surgical team and, consequently, the students' learning in real time, not delayed as previous studies had done. Our study shows that Tobii glasses 2 are considered a good tool for pointing out structures during the surgery, giving more precise “visual” instructions to the students, improving the precision of the novice surgeon and minimizing mistakes, and consequently improving the safety of the surgical patient, avoiding learning of complex surgical techniques. They are also a good didactic tool because they allow carrying out the learning based on gaze control, as much as with videos of the surgery with the gaze patterns of the surgeon registered during the real surgery, like being in the same operating room, thanks to the instant projection of foveal fixation. This possibility of pointing out with higher precision is strongly important to improve precision during surgery. Pucket and Baronia (18) carried out the first study that recorded the eye gaze patterns of an expert and a novice at the same time during live surgery. They described the correlation in the visual attitudes and movements (~85%) between the expert and the novice. Another study (9) described the authors' experiences with performing eye tracking for improving surgery training in the operating room. In our knowledge, no survey about the use of Tobii glasses during real surgery has been reported.

Video libraries have been used as a valid model for learning in laparoscopic surgery and orthopedics. In addition, it has been proposed to record videos from the head of the surgeon in order to capture their visual field. This has been attempted with the “Google Glass.” Adventure sports cameras provide high-quality videos, and their use has also been proposed (19). However, these videos do not have the visual key points during the performance of a surgery printed in their image. With the recent advances in the da Vinci “dual-console” systems and remote collaborative surgery, the demand to accurately represent (20) the point of view on the surgical screen is increasing. This need can be met by a novice's use of gaze-contingent technology and can improve the communication modalities available for surgeons of all grades (2). This is the main advantage of the application of eye-tracking technology in real time. For this reason, studies that explore the didactic potential of eye tracking in surgery have been carried out. In the literature review, we have found several studies (2, 5, 20–22) that analyzed the impact of the use of eye tracking in novices' learning. Three of them were performed in a laparoscopic surgical simulator. These studies (2, 22, 23) show that training based on visual control (watching where the expert surgeon fixes his eyes on) has better results than seeing only the recording from the surgeon's perspective (21) or receiving verbal instructions (2, 24). However, although the results of these studies are relevant, all were limited by the fact that they were performed in a simulator. Wilson et al. (22) reported that observation patterns obtained from the expert surgeon should be recorded during a real operation and then overhead on the registered surgical video, which would provide additional information to beginning surgeons about where the expert surgeon is focusing his/her attention on during every step of the operation. Besides, other studies (3, 10, 12, 17) analyzed the gaze patterns of surgeons and novices in the real operating rooms. Atkins et al. (12) used a “remote” eye tracker—which means a future analysis of the gaze, not in real time during surgery—and observed that there were differences between the gaze patterns of experts and residents.

On the other hand, there are studies that have suggested the use of eye tracking (ocular metrics) to assess an objective evaluation in medical (24, 25) and surgical education (2, 17, 26, 27) and also in high-stakes assessment analogously to other validated objective evaluations, such as the Operative Performance Rating System, the Objective Structured Assessment of Technical Skills (OSATS) (3), or the Global Operative Assessment of Laparoscopic Skills (GOALS) (17). Eye tracking could serve to evaluate skills because this technology allows perceiving not only the pattern of gaze but also the frequency of fixation and the time of permanence, which are used as measures of the importance of the perceived stimulus, as well as pupil dilation, a marker of the effort and concentration of the subject (3, 10, 12). Richstone et al. (15) showed that ocular metrics such as pupil size, blink rate, and fixation rate could be used as objective measures of surgical skill because they were able to classify experts and novices with great precision. The results of the study of Erridge et al. (16) suggested that experts focused on significant stimuli, had increased concentration, and had reduced mental workload. But also because the gaze patterns were different, as we have seen between experts and novices, so students could be assessed by checking that their patterns are more and more similar to those of the experts (17). Dynamic areas of interest reflected that the expert's eye gaze was able to differentiate expertise and the presence of unexpected adverse events (28).

The main limitation of our study is that the evaluation of this tool has been through answers of a survey. The ideal for future research would be to demonstrate this usefulness in terms of ease, speed, and safety through a randomized clinical trial where the usefulness of learning with and without this device is evaluated. Ideally, we would use the Tobii glasses 3 (https://www.tobiipro.com/es/products/tobii-pro-glasses-3/), which is the most modern version, not available when this study was carried out. Another aspect is that the total battery time is 120 min, and it is possible that an operation lasts longer than 120 min. In this case, it is possible to have another battery and change it during the procedure.

## Conclusion

The results of the current study, with high scores on the survey, allow us to verify the positive assessment of the usefulness of this eye-tracking device in the field of laparoscopic surgery as an objective learning tool for residents, improving their precision, thanks to the visual instructions, with the foveal vision of the surgeon projected in real time as a “pointer” controlled by sight. This technology minimizes the risk of mistakes, improves communication among members of the operating room, and also improves the learning of complex surgical techniques, resulting in increased patient safety during laparoscopy. It is a good didactic tool because it allows carrying out the learning based on gaze control, as much as with videos of the surgery with the gaze pattern of the surgeon registered during the real surgery, like being in the same operating room, thanks to the instant projection of foveal fixation. Finally, it could be a good tool to evaluate the residents' learning in an objective way, comparing the gaze patterns of experts and novices (the more similar, the better their surgical skill).

## Data Availability Statement

The original contributions presented in the study are included in the article/Supplementary Material, further inquiries can be directed to the corresponding author/s.

## Ethics Statement

The studies involving human participants were reviewed and approved by the Ethics Research Committee of our University and our Clinical University Hospital (No. 2015-10-5-HCUVA approved October 26, 2015). The patients/participants provided their written informed consent to participate in this study.

## Author Contributions

MS-F performed the laparoscopic procedure, wore the Tobii glasses during surgery, and revised the manuscript. FS-F and MG-M calibrated the device. EM-C wrote the manuscript and conducted the bibliographic research. All authors approved the final version of the manuscript.

## Conflict of Interest

The authors declare that the research was conducted in the absence of any commercial or financial relationships that could be construed as a potential conflict of interest.

## Supplementary Material

Supplementary Table 1 Analysis of studies using eye-tracking systems in laparoscopic surgery.

Supplementary Video 1 Link to the video of laparoscopic surgery in which the foveal fixation of the surgeon is marked by the Tobii glasses: https://youtu.be/9wTrB8Ssoec.
