# Supplementary material for: The Application of a System of Eye Tracking in Laparoscopic Surgery: A New Didactic Tool to Visual Instructions
Source: Front Surg. 2021 Jun 9;8:643611. doi: 10.3389/fsurg.2021.643611 (PMC8219847; doi:10.3389/fsurg.2021.643611)
Supplement: Supplementary Table 1 — Analysis of studies using eye-tracking systems in laparoscopic surgery. [file Table_1.DOCX]

**Supplementary Table.** Analysis of studies using eye-tracking systems in laparoscopic surgery

|  | Eye-tracking system | | Task | Participants | Results | | |
| --- | --- | --- | --- | --- | --- | --- | --- |
| Richstone et al. (2010) (7) | EyeLink II (head-mounted eye  tracker) | 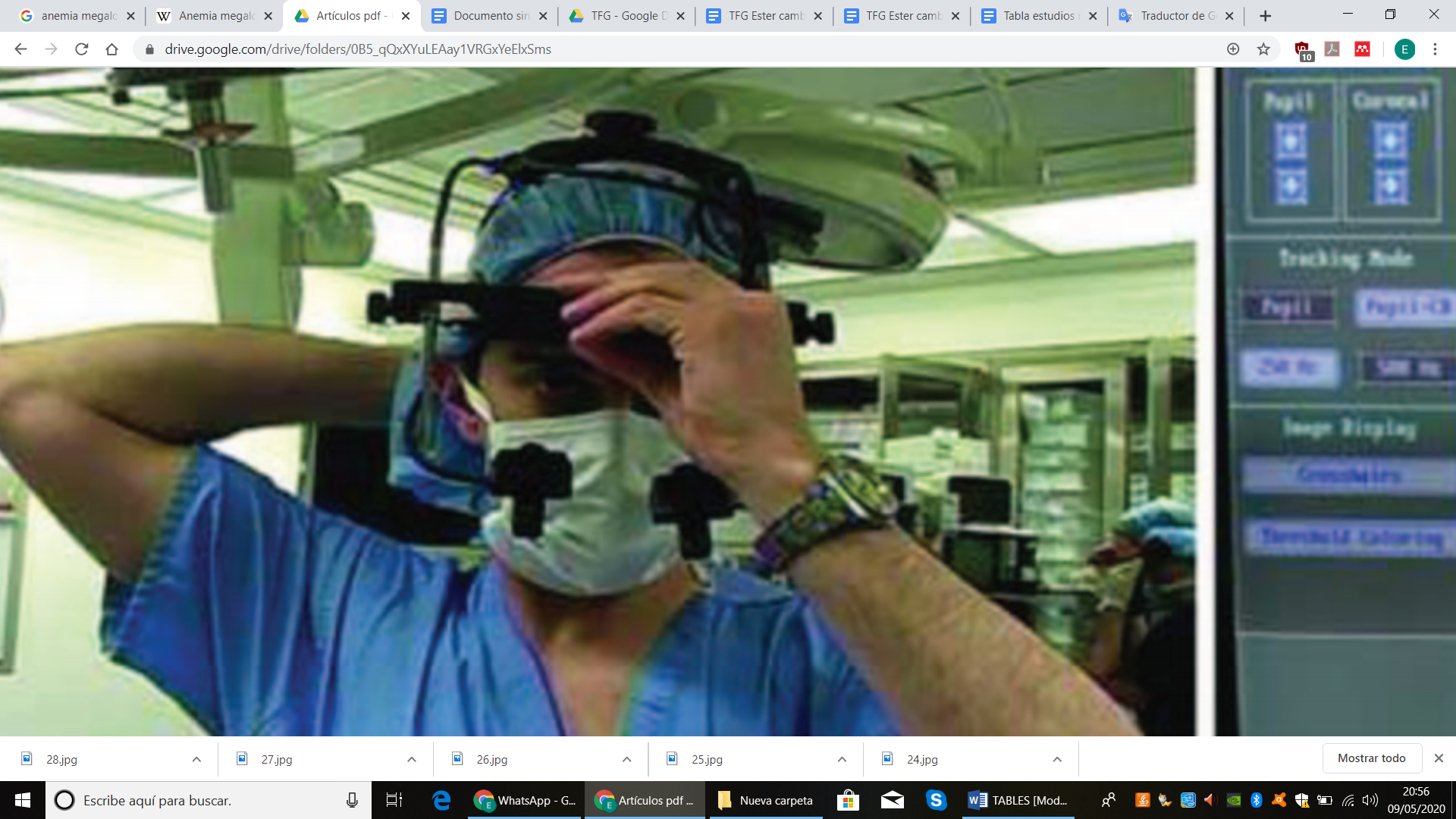 | Simulated and live laparoscopic surgery (laparoscopic renal surgery) | - 3 expert surgeons  - 18 non expert surgeon | **Pupillometry**  There were differences between expert and non expert surgeons (pupil size, blink rate and fixation rate). These data were related to the workload and the difficulty of the tasks. | | |
| Zheng, Jiang and Atkins (2015) (4) | Tobii 1750 eye-tracker (Tobii 1750, Tobii Technology,  Danderyd, Sweden). | 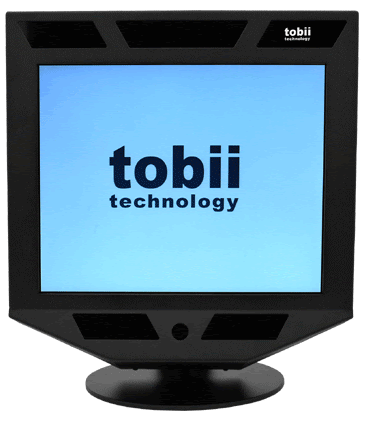 | Simulated laparoscopic surgery:  Subjects were required to perform a laparoscopic procedure that includes 9 subtasks. The subtasks could be divided into 3 types with different levels of task difficulty. | 14 right-handed university students with zero surgical experience. | **Pupillometry**  When the task difficulty was increased, task completion time increased. Meanwhile, the subjects’ peak pupil size also increased. | | |
| Zhang et al. (2017) (38) | Tobii glasses 2.0 | 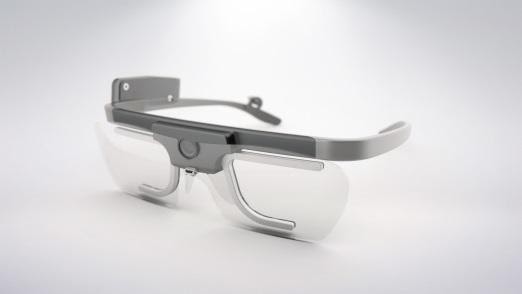 | Simulated laparoscopic surgery (laparoscopic cholecystectomy (LC) procedures in a virtual simulator) | -4 laparoscopic surgeons  -10 predoctoral students | **Pupillometry**  Significant differences were observed in pupil dilation between the three phases of laparoscopic surgery. This indicates that mental fatigue does markedly interfere with surgeons’ operating movements. | | |
| Di Stasi et al. (2016) (9) | Tobii glasses 2.0 (30Hz) | 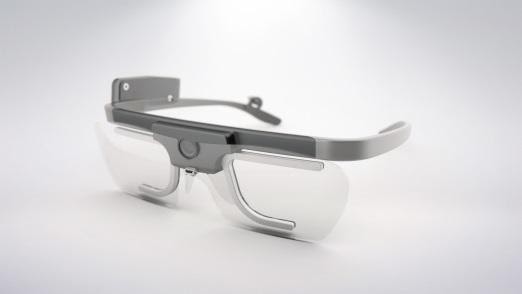 | Simulated laparoscopic surgery (3 virtual simulations of  laparoscopic exercises of increasing complexity level) | 18 surgical residents | **Gaze entropy**  Gaze entropy and velocity linearly increased with  increased task complexity (visual exploration pattern  became less stereotyped). These data showed that gaze metrics are a valid and reliable surgical task load index. | | |
| Law et al. (2004) (27) | ASL 504 remote eye tracker | 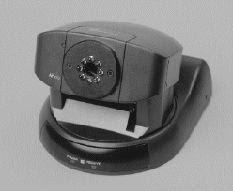 | Simulated laparoscopic surgery | - 5 experts  - 5 novices | **Gaze pattern**  Experts were quicker and generally committed  fewer errors than novices.  Novices needed more visual feedback of the tool position to complete the task, whereas experts tended to maintain eye gaze on the target while manipulating the tool. | | |
| Kocak et al. (2005) (28) | Saccadometer:  Cyclops Eye Trak saccadometer (Bertec Corporation, Columbus, Ohio) | 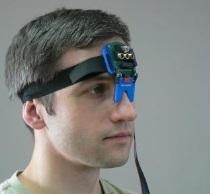 | Simulated laparoscopic surgery | - 8 expert surgeons  - 8 intermediate surgeons  - 8 novice surgeons | **Gaze pattern**  Expert surgeons moved their eyes less and spent more time at a certain point. | | |
| Wilson et al. (2010) (29) | Applied Science Laboratories Mobile Eye gaze registration system (ASL, Bedford, MA). | 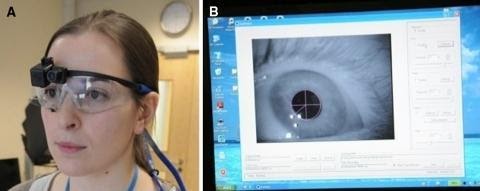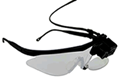 | Simulated laparoscopic surgery | - 8 experienced operators (right-handed)  - 6 novice operators (right-handed) | **Gaze pattern**  The experienced surgeons completed the task  significantly more quickly than novices, and experienced surgeons spent significantly more time fixating the target locations than novices, who split their time between focusing on the targets and tracking the tools. | | |
| Atkins, Tien, Khan, Meneghetti and Zheng. 2013 (real date: Jun 13 2012) (5) | Tobii 1750, 17-inch computer monitor (Tobii Technology AB,  Danderyd, Sweden) | 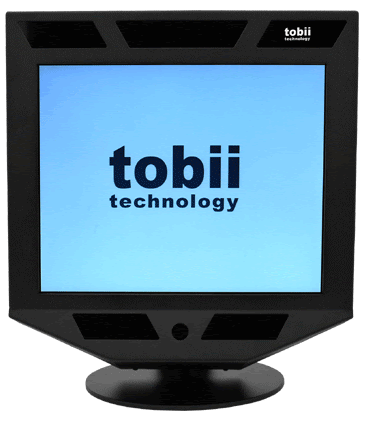 | Real laparoscopic surgery:  The viability of the use of eye-tracking technology in the real operating room was tested, in order to be able to implement it as a teaching tool. With this aim, 3 different eye-tracking systems were compared. | 3 eye-tracking systems:  - Tobii 1750 (50Hz)  - Tobii X50 (50Hz)  - Locarna PT-Mini head-mounted (30Hz) | It was concluded that the head mounted system (30Hz) did not have enough resolution, and the Tobii 1750 (50Hz) (high resolution ocular tracking device) had very restricted mobility, because it was incorporated in a monitor. In the end, they used the Tobii x50 (50Hz) device, which had high quality and was remote (it was separated from the monitor). | | |
|  | Tobii X50 (Tobii  Technology AB) | 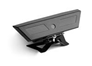 |  |  |  |  |  |
|  | The Locarna PT-Mini head-mounted eye-tracker | 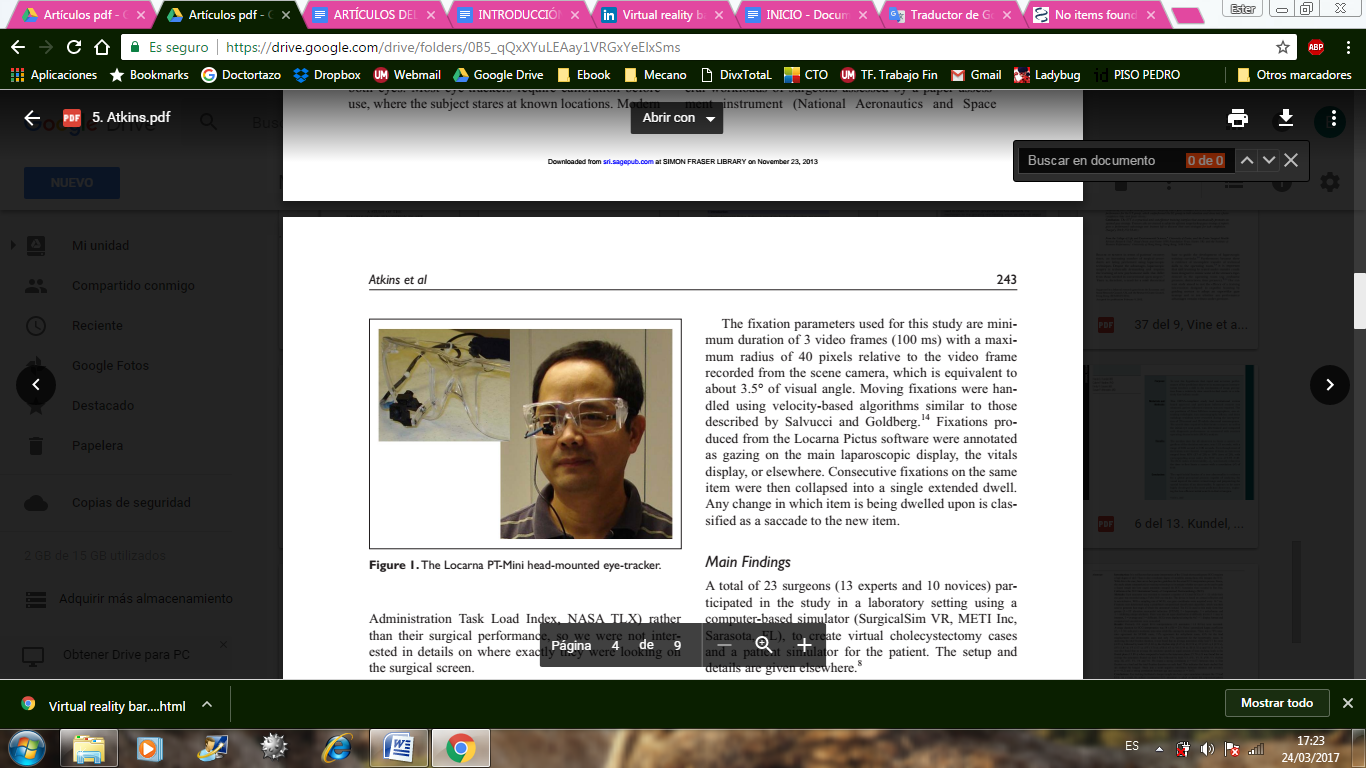 |  |  |  |  |  |
| Khan, Tien, Atkins, Zheng, Panton and Meneghetti (Jun 26 2012) (32) | Tobii X50 (Tobii  Technology AB) | 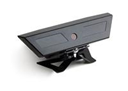 | Real laparoscopic surgery:  16 laparoscopic cholecystectomies were recorded with the gaze pattern of the surgeon. Afterwards, surgeons and novices watched the videos, also tracking their gaze. | -2 expert surgeons  -Junior residents | **Gaze pattern**  The expert surgeons developed an ability to scan over surgical sites using a replicable strategy over different trials. In contrast, novice surgeons did not develop a stable strategy and had a lower chance to copy the expert’s visual strategy. | | |
| Tien, Atkins, Jiang, Khan, Zheng. (2013) (35) | Tobii X50 (Tobii  Technology AB) | 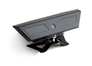 | Real laparoscopic surgery: Eye tracking data of expert surgeons were recorded during 8 laparoscopic cholecystectomy cases. Afterwards, operating surgeons and trainee residents  were invited to have their gaze tracked while watching the captured laparoscopic video feed from the cases performed previously. | -Expert surgeons  -Residents | **Gaze pattern**  There were differences between gaze patterns of experts and residents. Watching his own case, the operating surgeon had fewer mismatches than when the case was watched by residents. Mismatches dropped more in frequency during precision surgical tasks such as isolation and severance of the cystic duct and artery. | | |
| Pucket and Baronia (2016) (34) | EyeGuide® Mobile Tracker headset and pack (Grinbath, Lubbock, TX) | 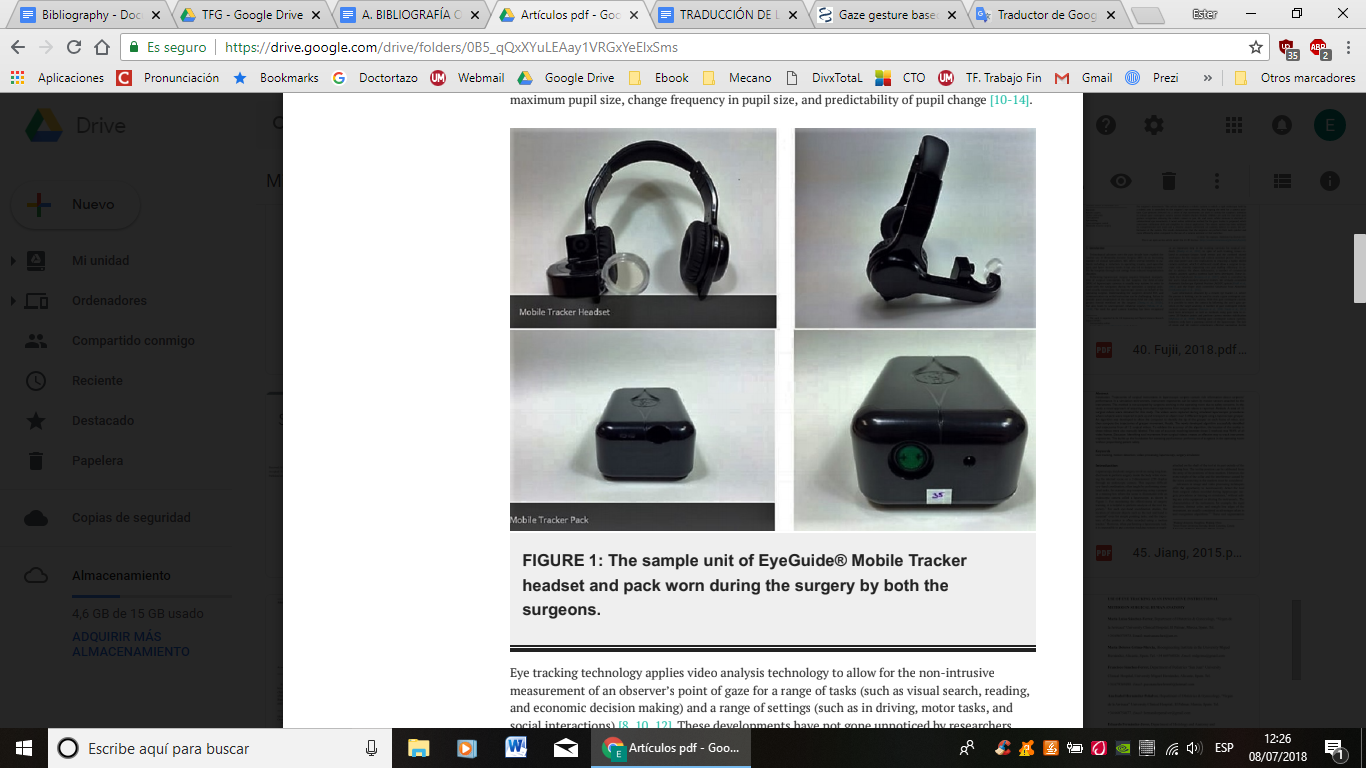  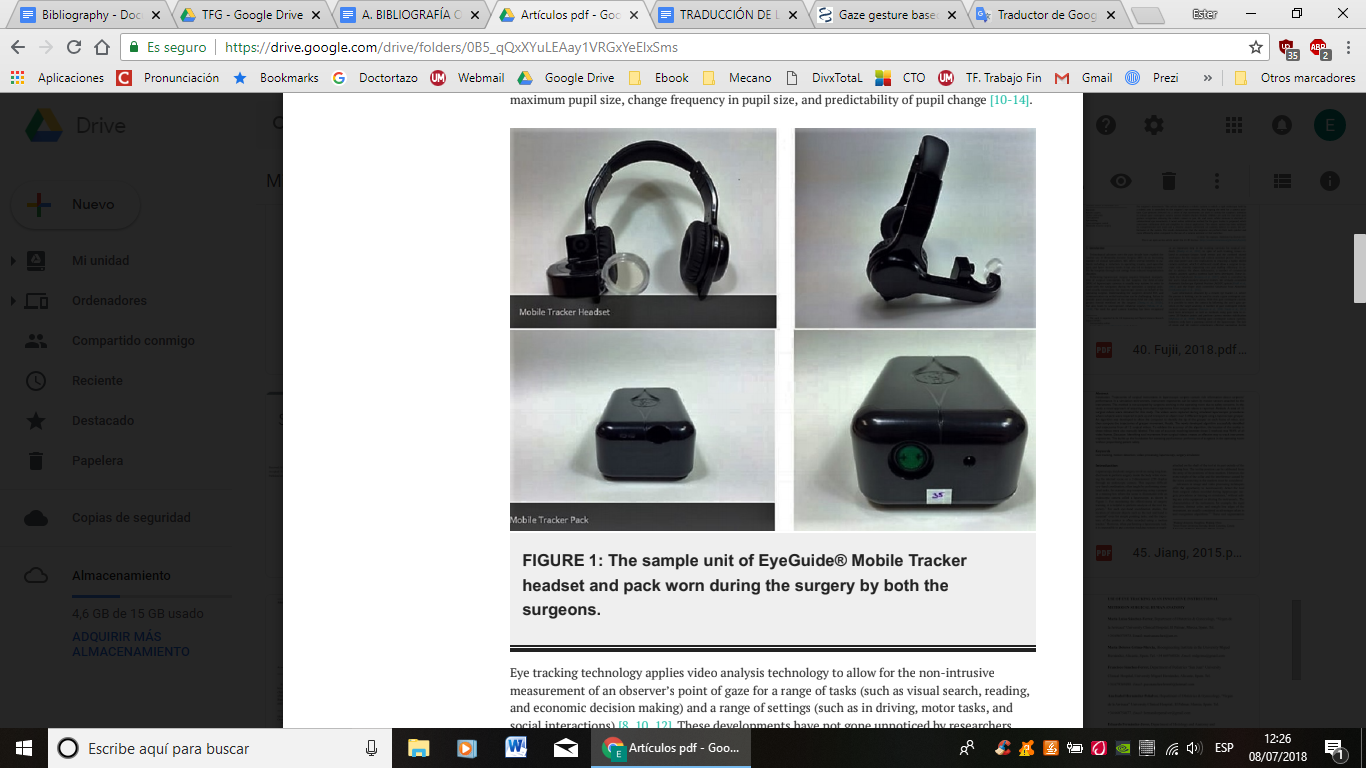 | Real laparoscopic surgery: Participants performed a live laparoscopic cholecystectomy, while simultaneously wearing  visual tracking devices. | -1 expert surgeon  -1 chief surgical resident | **Gaze pattern**  The visual attitudes and movements correlated approximately 85% between an expert surgeon and a chief surgical resident. The expert demonstrated higher fixation frequency, dwell time on the operative site during the application of clips and during dissection of critical view of safety, and dwelled more on the sterile field during the removal of the gallbladder out of the port site. We conclude that simultaneous deployment of visual tracking during live laparoscopic surgery is a possibility. | | |
| Sánchez Ferrer et al. (2017) (25) | Tobii glasses 2 | 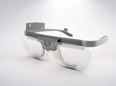 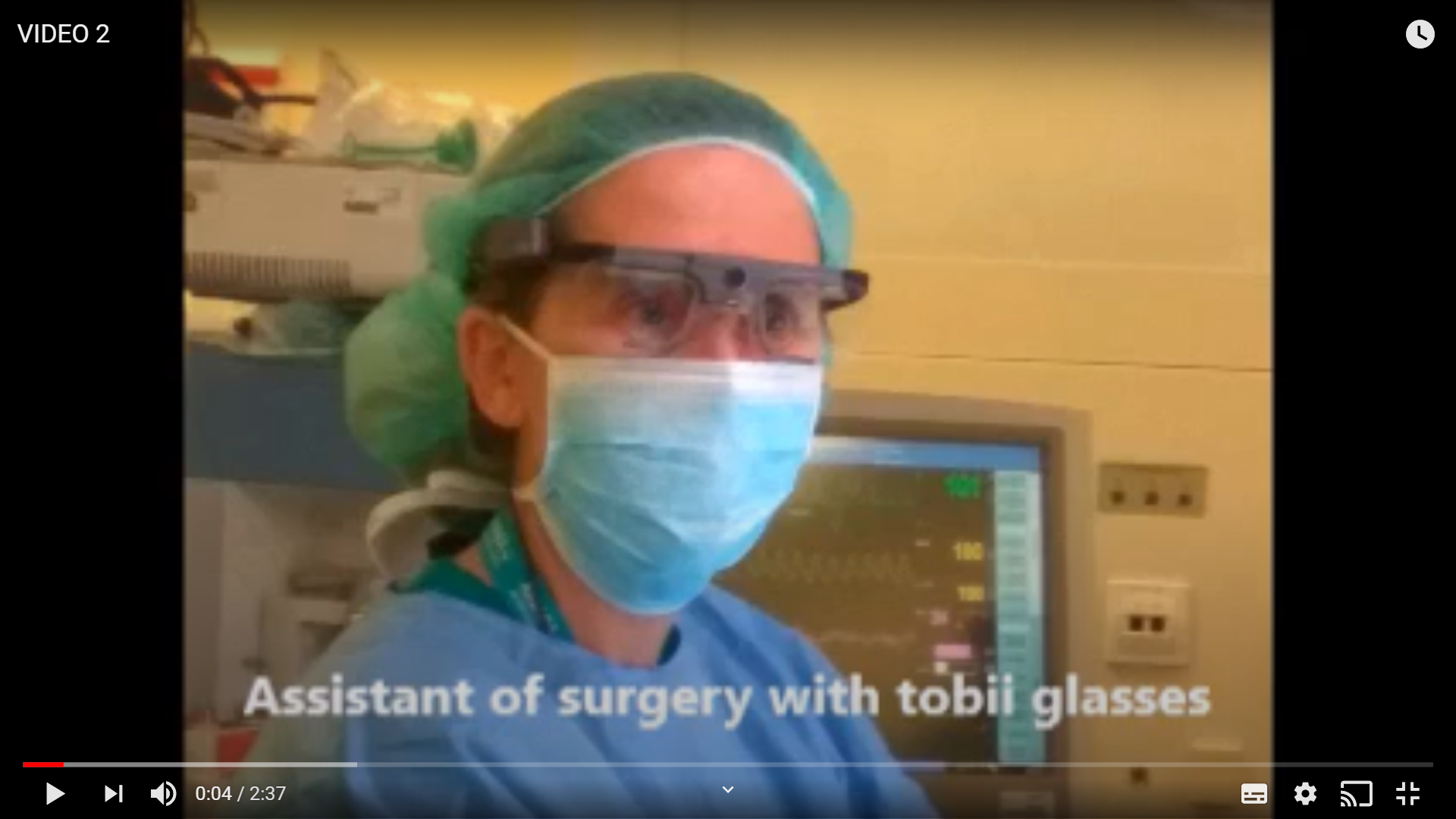 | Real laparoscopic surgery:  It was recorded a right adnexectomy by laparoscopy for ovarian cyst. The gaze pattern of the surgeon was showed in a monitor as a red point. | 1 expert surgeon | **Gaze pattern**  It was concluded that Tobii glasses 2 were comfortable (they are remote), easy to use, had enough quality to record in the operating room (50Hz), and could be used as a "third hand" and it would allow to do gaze control during the surgery. | | |
| Erridge et al. (2018) (33) | Eye-tracking glasses, model 1.4; SMI (SensoMotoric Instruments), Teltow, Germany. |  | Real laparoscopic surgery:  Laparoscopic Roux-en-Y gastric bypass (LRYGB) for obesity was recorded from participants while performing the procedures. Afterwards, the videos of the surgery were analysed. | -8 expert  -12 junior | **Gaze pattern**  These results suggest that experts display more focused attention to significant stimuli, alongside experiencing a reduced mental workload and having increased concentration. This has the potential for future use in validation of surgical skill in high-stakes assessment. | | |
| Fichtel et al (2019) (39) | Remote eye tracker (SMI, Teltow, Germany) attached to the monitor (30Hz) | 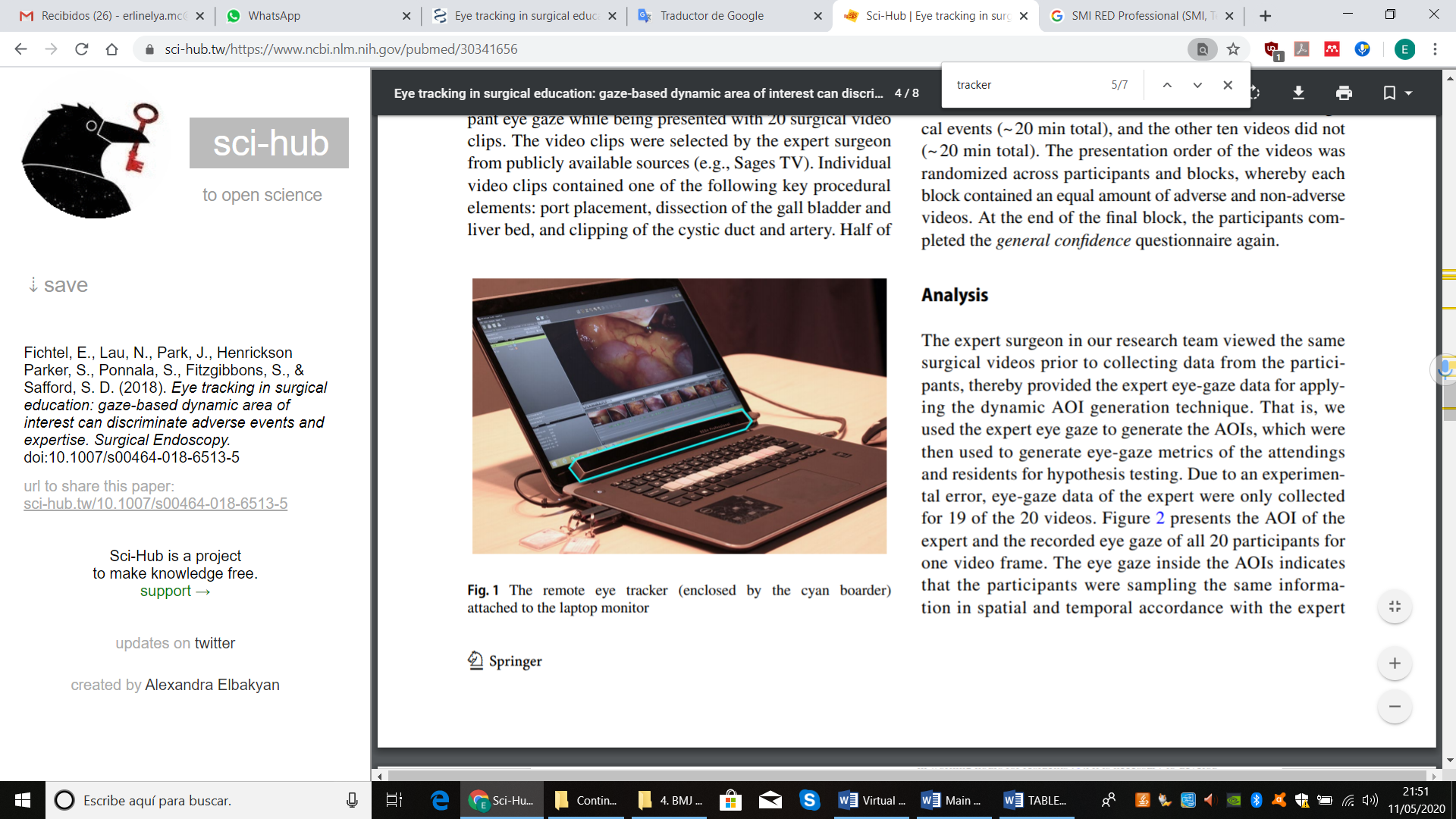 | Real laparoscopic surgery:  Participants were asked to see laparoscopic cholecystectomy videos (from publicly available sources) while recording their gaze pattern. This was compared with the areas of interest (AOIs) of an expert surgeon | -10 expert  -10 surgical resident | **Gaze pattern**  Dynamic AOIs reflected the expert eye gaze was able to differentiate expertise, and the presence of unexpected  adverse events. | | |
| Wilson et al. (2011) (30) | Applied Science Laboratories Mobile Eye gaze registration system (ASL; Bedford, MA) | 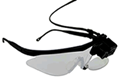 | Simulated laparoscopic surgery:  Training consisted of ten repetitions of the ‘‘eye-hand coordination’’ task from the LAP Mentor VR laparoscopic surgical simulator, while receiving instruction and video feedback (specific to each treatment condition). | 30 medical trainees with no laparoscopic experience were divided randomly into one of three treatment groups. | **Education** | | |
|  |  |  |  |  | 3 groups of training:   - 10 gaze trained (they saw the surgeon gaze pattern and tried to imitate it) (GAZE) - 10 movement trained (control of movements)(MOVE) - 10 discovery learning/control (DISCOVERY) | | The best learning was obtained by the group based on gaze control. |
| Chetwood et al. (2012) (24) | Tobii 1750, 17-inch computer monitor (Tobii Technology AB,  Danderyd, Sweden) | 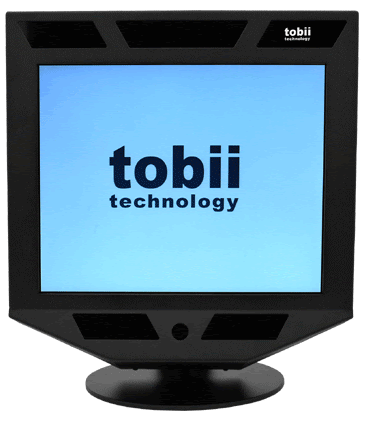 | Simulated laparoscopic surgery:  Subjects performed the task receiving instructions from an expert at the same time, different according to the group. | 28 subjects with varying levels of operative experience and proficiency laparoscopic tasks. | **Education** | | |
|  |  |  |  |  | 3 groups with different instructions:   - Verbal queues (V) - A cursor reflecting supervisor’s eye-gaze (E) - Both (VE). | Completion times and number of errors were significantly reduced when eye-gaze instruction was employed (VE, E). In addition, the time taken for the subject to correctly focus on the target (latency) was significantly reduced. | |
| Vine et al. (2012) (31) | Mobile Eye gaze  registration system (Applied Science Laboratories,  Bedford, ASL, MA), | 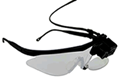 | Simulated laparoscopic surgery:  Subjects performed 50 learning trials of a laparoscopic training task. There were a discovery-learning group and a gaze-training group. At the end, both groups took part in a non-delayed retention test (to assess learning) and a stress test (under social evaluative threat) with a normal view of the scene. | 27 novices (who had no laparoscopic training) | **Education** | | |
|  |  |  |  |  | 1. groups:  - Gaze-training (GT): it was used a software designed to guide expert-like gaze strategies by highlighting the key locations on the monitor screen. - Learning-discovery (LD): they had a normal, unrestricted view of the scene on the monitor screen.   There were neither verbal nor written instructions. | Gaze-training group had faster completion time and fewer errors at the retention and stress test. | |
| Jiang, Zheng and Akins (2015) (37) | Tobii 17/50  eye-tracker, 17-inch computer monitor | 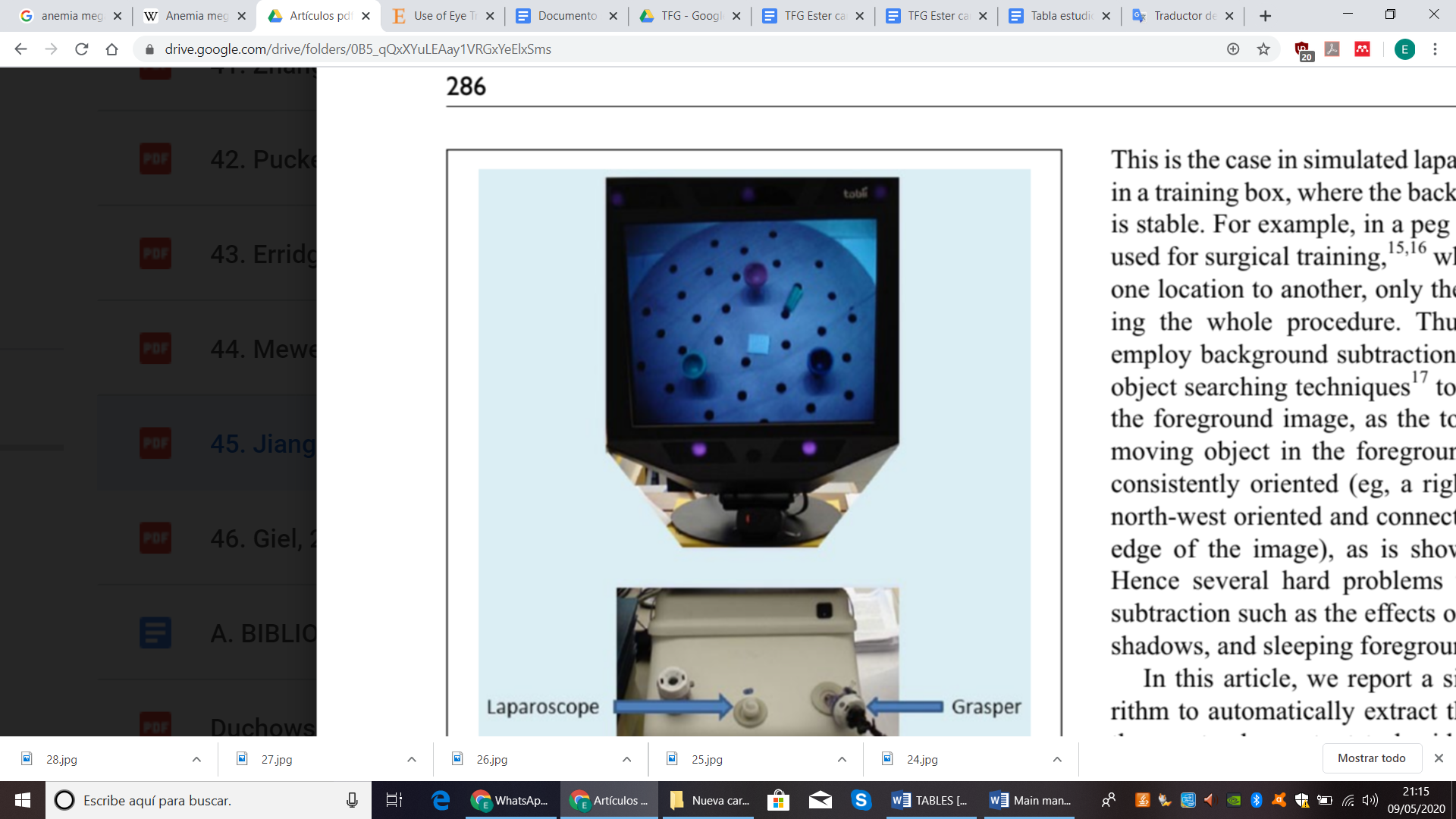 | Simulated laparoscopic surgery:  Subjects were required to pick up and transport an object over 3 different targets using a laparoscopic grasper. An algorithm was developed to allow the computer to identify the tip of the grasper on each frame of video, and then compute the trajectories of grasper movement.  A total of 12 task videos (the first trial of each participant)  were processed by the algorithm to output the tooltip positions. | 12 participants | **Evaluation**  To validate the accuracy of the algorithm, the location of the tooltip in these videos were also manually labelled. The rate of accurate matching between these 2 methods was 98.4% of all video frames.  Identifying tool movement from surgical videos creates an effective way to track instrument trajectories. This builds up the foundation for assessing psychomotor performance of surgeons in the operating room without jeopardizing patient safety. | | |
